# Identification of polysaccharide capsules among extensively drug-resistant genitourinary *Haemophilus parainfluenzae* isolates

**DOI:** 10.1038/s41598-019-40812-2

**Published:** 2019-03-14

**Authors:** Aida González-Díaz, Fe Tubau, Miguel Pinto, Yanik Sierra, Meritxell Cubero, Jordi Càmara, Josefina Ayats, Paula Bajanca-Lavado, Carmen Ardanuy, Sara Marti

**Affiliations:** 1 Microbiology Department, Hospital Universitari de Bellvitge, Universitat de Barcelona-IDIBELL, Barcelona, Spain; 2 CIBER de Enfermedades Respiratorias (CIBERes), Instituto de Salud Carlos III, Madrid, Spain; 3 Bioinformatics Unit, Department of Infectious Diseases, National Institute of Health, Lisbon, Portugal; 4 Haemophilus influenzae Reference Laboratory, Department of Infectious Diseases, National Institute of Health, Lisbon, Portugal

## Abstract

The human commensal *Haemophilus parainfluenzae* is emerging as an opportunistic multidrug-resistant pathogen. The objectives of this work were to characterise a new capsular operon of extensively drug-resistant (XDR) *H. parainfluenzae* clinical isolates and study their resistance mechanisms using whole-genome sequencing. All strains were resistant to: ß-lactams, via amino acid changes in PBP3 (S385T, I442F, V511A, N526K and V562I); quinolones, by alterations in GyrA (S84F and D88Y) and ParC (S84F and S138T); chloramphenicol, through the presence of *catS*; macrolides, via the presence of *mel* and *mef(E)*-carrying MEGA element; and tetracycline, through the presence of *tet(M)* and/or *tet(B)*. Phylogenetic analysis revealed high genomic diversity when compared to the *H. parainfluenzae* genomes available on the NCBI, the isolates from this study being closely related to the Swiss XDR AE-2096513. A full capsular operon showing homology to that of *H. influenzae* was identified, in accordance with the observation of a capsular structure by TEM. This study describes for the first time a capsular operon in *H. parainfluenzae*, a major determinant of pathogenicity that may contribute to increased virulence in XDR clinical isolates. Moreover, phylogenetic analysis suggests the possible spread of an XDR-encapsulated strain in Europe.

## Introduction

The members of the genus *Haemophilus* are pleomorphic and fastidious Gram-negative coccobacilli that require supplementary factors for *in vitro* growth, particularly V-factor (β-nicotinamide adenine dinucleotide, NAD) and/or X-factor (haem)^1,2^. These nutritional requirements are essential in dividing this genus into three groups: the haem-dependent *H. influenzae* group (*H. influenzae, H. haemolyticus* and *H. aegyptius*); the haem-independent *H. parainfluenzae* group (*H. parainfluenzae, H. parahaemolyticus, H. paraphrohaemolyticus, H. pittmaniae* and *H. sputorum*); and the group containing *H. ducreyi* that requires haem, but not NAD for growth^1^. As a representative of the haem-independent group, *H. parainfluenzae* is characterised by its ability to synthesise haem, while depending on the presence of NAD in the environment for growth^1^.

*H. parainfluenzae* is part of the human oropharyngeal and genitourinary microbiota and is increasingly recognised as an opportunistic pathogen causing invasive, chronic or recurrent diseases, including respiratory tract infections^3^, meningitis^4^, endocarditis and pericarditis^5^, bone and joint infections^6^, and arthritis^7^. Recent reports have also linked this pathogen to genitourinary and sexually transmitted infections^8–10^, with one study observing unexpectedly high genital carriage in pregnant women that was frequently associated with antibiotic resistance traits^8^. In urethral exudates from men with acute *Haemophilus* spp. urethritis, *H. parainfluenzae* was observed to be six times more frequent than *H. influenzae*^10^. Likewise, urinary tract infections in children have shown differences with gender, with *H. influenzae* being the main species identified in girls and *H. parainfluenzae* in boys presenting urinary tract abnormalities, such as malformation, gross reflux or bladder dysfunction^11^. Moreover, *H. parainfluenzae* urethritis has also been described in men who have sex with men (MSM), highlighting the potential role of this microorganism in causing sexually transmitted diseases (STD)^9^.

Virulence factors play a crucial role in the invasion and infection process. Although the capsule has not yet been described in *H. parainfluenzae*, it is one of the most important virulence factors in *H. influenzae* and has been used for vaccine development. To date, six different capsular serotypes have been described for *H. influenzae* (a to f). The genes encoding the capsule are located in the *cap* locus, which is divided into three different regions. Region I contains four common genes (*bexDCBA*) that are responsible for the translocation of the capsular polysaccharides. Genes belonging to region II are serotype-specific and are associated with polysaccharide biosynthesis. Finally, region III contains two genes (*hcsAB*) that are involved in post-polymerisation steps^12^.

Along with virulence factors, antimicrobial resistance is also important during the course of infection and identifying such resistance is required to apply correct antimicrobial treatment. *H. parainfluenzae* is often resistant to β-lactam antibiotics^13^ and although resistance to fluoroquinolones and macrolides is still uncommon, isolates presenting reduced susceptibility to these antimicrobial agents are being increasingly reported^14^. In 2011, a multidrug-resistant (MDR) *H. parainfluenzae* clinical isolate, presenting resistance to quinolones, tetracycline and co-trimoxazole, was reported in a patient with prostatitis in Spain^15^. Two years later in Switzerland, a case study of an MSM man co-infected with a pan-susceptible *Neisseria gonorrhoeae* isolate and an extensively drug-resistant (XDR) *H. parainfluenzae* strain was described^16^. XDR microorganisms are defined as resistant to at least one agent in all but two or fewer antimicrobial categories^17^ and are a significant cause for concern. Our study aimed to describe for the first time a capsule in four XDR *H. parainfluenzae* strains and fully characterise their molecular antibiotic resistance mechanisms.

## Results

### Characterisation of antimicrobial resistance

Three isolates were resistant to all the tested β-lactams (ampicillin, cefuroxime, cefotaxime, cefepime, amoxicillin/clavulanic acid and ceftriaxone), except for the carbapenems (imipenem and meropenem). A single isolate remained susceptible to amoxicillin/clavulanic acid and ceftriaxone. All the isolates were also resistant to chloramphenicol, macrolides, quinolones and co-trimoxazole, only remaining susceptible to rifampicin (Table 1).

**Table 1:** Strain data, MICs and molecular resistance mechanisms.

Isolate	Sex	Age	Source	ß-lactams										Chloramphenicol	
				AMP	AMC	CXM	CRO	CTX	FEP	IPM	MEM	PBP3		CHL	
**HUB11505**	**M**	**25**	**Urethral**	**4**	2/1	**>8**	≤0,12	**0,5**	**0.5**	0,25	≤0,25	S385T, I442F, V511A, N526K, V562I		**>8**	*catS*
**HUB12345** ^**a**^	**F**	**38**	**Vaginal**	**>4**	**>4/2**	**>8**	**0,25**	**1**	**2**	0,5	≤0,25	K276N, A307N, V329I, S385T, I442F, V511A, N526K, V562I		**>8**	*catS*
**HUB12445** ^**a**^	**F**	**38**	**Endocervical**	**>4**	**>4/2**	**>8**	**0,25**	**1**	**2**	0,5	≤0,25	K276N, A307N, V329I, S385T, I442F, V511A, N526K, V562I		**>8**	*catS*
**HUB 12640**	**M**	**35**	**Preputial**	**4**	**4/2**	**>8**	**0,25**	**1**	**2**	0,5	≤0,25	K276N, A307N, V329I, S385T, I442F, V511A, N526K, V562I		**>8**	*catS*
**Isolate**	**Macrolides**				**Quinolones**					**Tetracycline**			**Cotrimoxazole**		**Rifampicin**
	**ERY**	**AZM**	**L22**	**MEGA**	**CIP**	**LVX**	**GyrA**	**ParC**	**ParE**	**TET**			**SXT**	**DHFR**	**RIF**
**HUB11505**	**>16**	**>4**	A69S	*mef(E)*	**>2**	**>4**	S84F, D88Y	S84F, S138T, M198L	D420N, A451S	**>4**	*tet(M)*	*tet(B)*	**>2/38**		1
**HUB12345** ^**a**^	**>16**	**>4**	A69S	*mef(E)*	**>2**	**>4**	S84F, D88Y	S84F, S138T, M198L	D420N, A451S	**>4**	*tet(M)*		**>2/38**	I95L	0.5
**HUB12445** ^**a**^	**>16**	**>4**	A69S	*mef(E)*	**>2**	**>4**	S84F, D88Y	S84F, S138T, M198L	D420N, A451S	**>4**	*tet(M)*		**>2/38**	I95L	0.5
**HUB12640**	**>16**	**>4**	A69S	*mef(E)*	**>2**	**>4**	S84F, D88Y	S84F, S138T, M198L	D420N, A451S	**>4**	*tet(M)*	*tet(B)*	**>2/38**		0.5

MICs for each antibiotic are expressed in mg/L. Resistance based on the EUCAST criteria for *H. influenzae* is marked in bold. Resistance genotype refers to amino acid changes and acquired resistance mechanisms for ß-lactams, macrolides, quinolones, tetracycline and co-trimoxazole.

AMP, ampicillin; AMC, amoxicillin/clavulanic acid; CXM, cefuroxime; CRO, ceftriaxone; CTX, cefotaxime; FEP, cefepime; IPM, imipenem; MEM, meropenem; CHL, chloramphenicol; ERY, erythromycin; AZM, azithromycin; CIP, ciprofloxacin; LVX, levofloxacin; TET, tetracycline; SXT, co-trimoxazole; RIF, rifampicin.

^a^Indicates isolates from the same patient obtained on the same day.

The mechanisms of antimicrobial resistance were detected *in silico* (Table 1). Resistance to β-lactams was associated with amino acid substitutions in the penicillin-binding protein (PBP3). Five substitutions were present in all the strains [S385T, I442F, V511A, N526K and V562I], while three additional changes [K276N, A307N and V329I] were only present in HUB12435, HUB12445 and HUB12640. The presence of β-lactamase was not detected in any of the isolates. Fluoroquinolone resistance was attributed to amino acid substitutions in DNA gyrase (GyrA) [S84F and D88Y] and in DNA topoisomerase IV subunit A (ParC) [S84F and S138T] and subunit B (ParE) [D420N and A451S]. Macrolide resistance was conferred by the amino acid substitution A69S in the 50S ribosomal protein L4, encoded by the *rplD* gene, and by the acquisition of the MEGA element containing *mel* and *mef(E)*. Tetracycline resistance was attributed to the *tet(M)* gene located upstream of the MEGA element, as described previously for *H. parainfluenzae*^18^. Resistance to chloramphenicol was linked to the presence of an acetyltransferase encoded by the *catS* (CP015430.1:976644-977267) gene. Moreover, the HUB11505 and HUB12640 strains also acquired the *tet(B)* gene (NC_015964:1166619-1167824).

### Phylogenetic diversity

**Figure 1:**
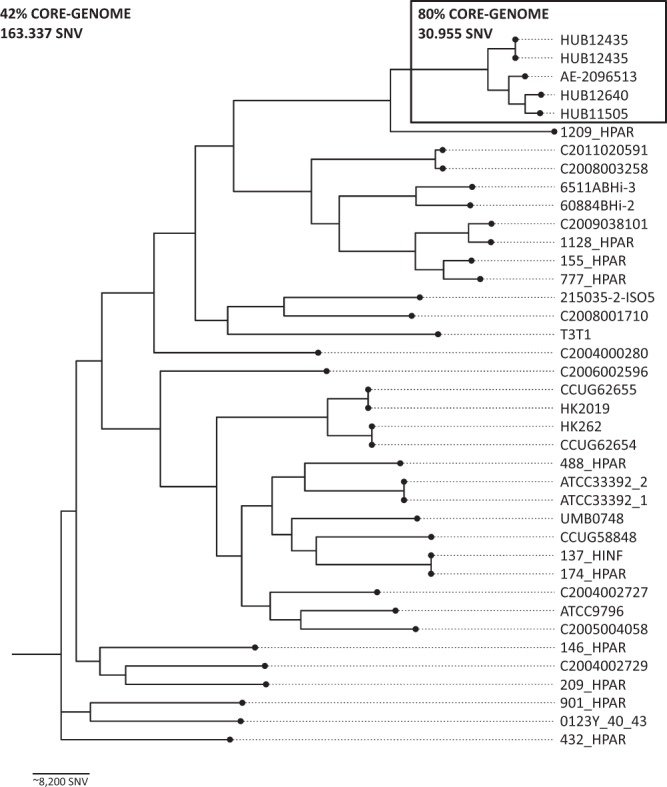
Phylogenetic relationship between the *H. parainfluenzae* strains. The 35 *H. parainfluenzae* genomes available on the NCBI database and the four XDR strains isolated in this study are presented in a core-single nucleotide variant (SNV) phylogenetic tree. Distance between the bacterial strains is represented in SNVs.

Phylogenetic analysis involved the 35 *H. parainfluenzae* genomes that were available on the NCBI database, including the *H. parainfluenzae* strain T3T1, which has a fully closed genome and was used as a reference strain for the phylogenetic analysis (Fig. 1). Overall, high intraspecies diversity was found, with an estimated core genome of around 42% and a total of 163,337 single nucleotide variants (SNVs). Despite this genetic diversity, the *H. parainfluenzae* strains isolated in this study were closely related (80% core genome and 30,955 SNVs) to an XDR strain from Switzerland (AE-2096513)^16^. Strains HUB12345 and HUB12445, isolated from a single patient, were genetically indistinguishable

### New capsular operon related to that of *H. influenzae* serotype c

A similar capsular operon was detected in all the strains, with a total length of 14,678 bp and 10 predicted ORFs. However, the capsular operon of HUB11505 presented a *pcsB* gene that was truncated at nucleotide position 5,613 by the insertion of an IS4 family transposase ISVsa5 previously identified in *Haemophilus ducreyi* (NZ_CP015434.1:125637-126845).

**Figure 2:**
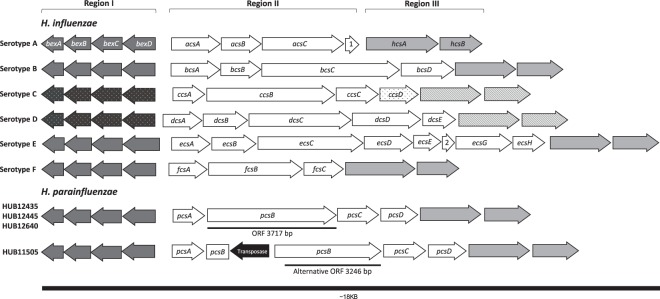
Genetic composition of *H. influenzae* and *H. parainfluenzae* capsular operons. Genes marked in dark grey belong to region I, while those from region III are marked in light grey. The sequences of *H. influenzae* serotypes c and d are not available on NCBI (genes represented by white dots in region I and genes represented by white stripes in region III). The *ccsD* gene represented by black dots has a 1-bp deletion, resulting in an interrupted gene. In the HUB11505 strain, *pcsB* is interrupted by a transposase, but there is an alternative ORF that has 471 fewer bp than that of HUB12435, HUB12445 and HUB12640. 1. ascD. 2. ecsF.

Figure 2 describes the genetic composition of the six *H. influenzae* and *H. parainfluenzae* capsular operons. A new name has been designated to each gene from the variable region II in *H. parainfluenzae*: *pcsABCD*. Regions I and III from serotypes c and d were not available on the NCBI database and hence, those genes were not included in the analysis. Supplementary Table 1 present the identity between each gene/protein from *H. parainfluenzae* and from the *H. influenzae* serotypes available on the NCBI database. Region I was the most conserved among the serotypes, with a high protein identity (>90%). Region III was also conserved, but the protein identity was lower than that of region I. The serotype-specific proteins from region II showed no identity with the new capsular operon, except for the genes *fcsA* (serotype f)*, ccsA, ccsC* and *ccsD* (serotype c). Although *ccsD* and *pcsD* presented high identity, *ccsD* from the NCBI database (HQ651151) had a 1-bp deletion at position 34 that resulted in a truncated protein.

### Visualisation of capsular polysaccharides

**Figure 3:**
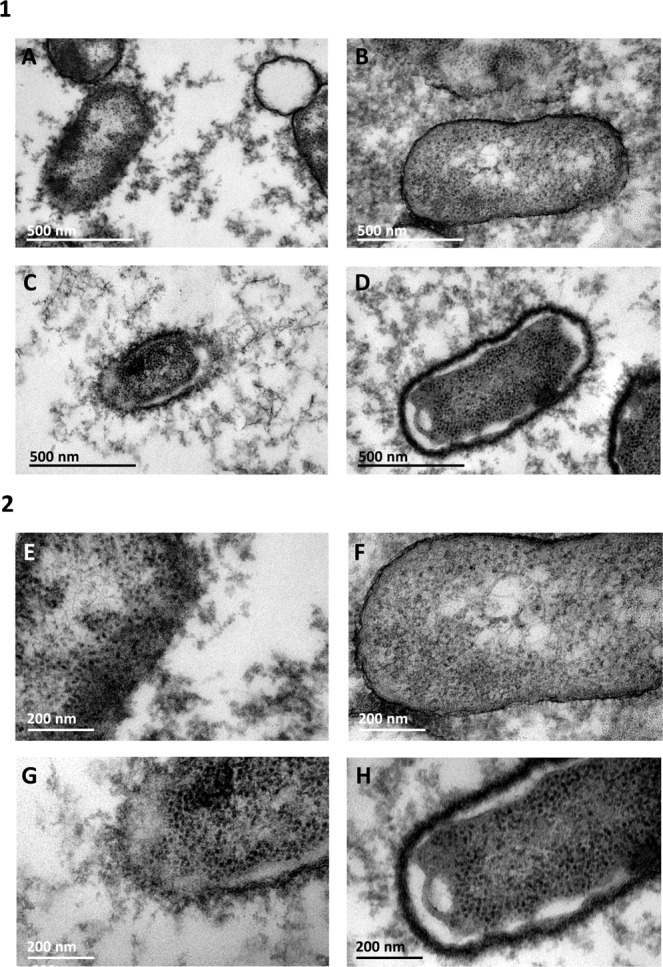
Capsule visualisation by TEM. (**A,E**) *H. influenzae* serotype b. (**B,F**) Non-encapsulated *H. parainfluenzae*. (**C,G**) Strain HUB12445. (**D,H**) Strain HUB11505. (1) Images acquired at a magnification of 120,000x. (2) Images acquired at a magnification of 200,000x.

Capsular polysaccharides were visually examined under the TEM, using *H. influenzae* serotype b and non-encapsulated *H. parainfluenzae* (*bexA-*negative) as controls. Cellular visualisation (Fig. 3) revealed that the non-encapsulated *H. parainfluenzae* was surrounded by a defined bacterial cell membrane, which was significantly thinner than that of the encapsulated *H. influenzae* serotype b and the *H. parainfluenzae* strain HUB12445. Strain HUB11505, with a truncated *pcsB* gene, was also surrounded by a diffuse structure that could be classified as an intermediate between that of the encapsulated *H. influenzae* serotype b and the non-encapsulated *H. parainfluenzae*.

## Discussion

*H. parainfluenzae* is a human commensal responsible for a large range of human infections, including genitourinary infections. The putative role of this pathogen in STDs is a cause for concern. In this paper, we describe for first time the capsular operon of four XDR *H. parainfluenzae* clinical isolates from three patients with urogenital infections, two males presenting urethritis and a female with vaginal discharge due to intra uterine device implantation.

The accumulation of many resistance mechanisms to several antimicrobial agents (ß-lactams, chloramphenicol, macrolides, quinolones, tetracycline and co-trimoxazole) leaves few available therapeutic options. Although β-lactam resistance is mainly due to the presence of β-lactamases^19,20^, the resistance in our strains was only associated with amino acid substitutions in the PBP3. These changes have been previously described in an *H. parainfluenzae* clinical isolate classified as a β-lactamase-negative ampicillin-resistant isolate (gBLNAR) from the III-like group^13^. Strain HUB11505 was susceptible to amoxicillin/clavulanic acid and ceftriaxone, possible due to the absence of substitutions K276N, A307N and V329I, which have already been reported^16^, but not conclusively proven to confer resistance to β-lactams. The analysis of the quinolone resistance-determining region (QRDR) of *gyrA*, *parC* and *parE* also revealed mutations that have been previously described^21^. The substitutions in DNA gyrase (S84F and D88Y) have been reported to be essential in the interaction between DNA and fluoroquinolones in *H. influenzae*^22^. Both substitutions, together with S84F in ParC, are the most common changes associated with fluoroquinolone resistance in Gram-negative bacteria. The ParC substitutions S84F and D420N, together with A451S in ParE have also been linked to high-level fluoroquinolone resistance in *H. parainfluenzae*, but their involvement in resistance has not been demonstrated^15^. The substitutions S138T and M198L have also been previously described^8^ outside the QRDR region in ParC, which might be involved with fluoroquinolone resistance^14^. Although the substitution A69S in the L4 protein has already been found in macrolide-resistant isolates^23^, a recent report also identified this mutation in susceptible strains, suggesting that it alone might not confer resistance to macrolides^14^. However, the decrease in susceptibility could also be explained by the presence of the macrolide efflux genetic assembly (MEGA) element, which is present in different bacteria and was found for the first time in an *H. parainfluenzae* isolate reported in 2013 in Switzerland^18^. This element contains the *mel* and *mef(E)* genes, which encode a dual efflux pump that confers resistance to 14- and 15-membered lactone ring macrolides. Related to co-trimoxazole resistance, the I95L substitution in dihydrofolate reductase (DHFR) has been proposed to be involved in resistance^24^ and could be a homologue for the I100L substitution found in *S. pneumoniae*^25^. However, only two of the strains investigated in this study presented this change, suggesting that additional mechanisms might contribute to this resistance. Finally, all the strains presented *catS*, an acquired mechanism of resistance to chloramphenicol from *H. ducreyi*, suggesting the possible horizontal transfer of genetic material between strains from different species.

As observed in Fig. 1, *H. parainfluenzae* presents high intraspecies diversity with a low percentage of core genome and a high number of SNVs, in accordance with previous epidemiological studies performed by PFGE in our hospital (data not shown). The high genome plasticity in this species facilitates the exchange of genetic material with other microorganisms, enabling this species to act as a genetic reservoir of antimicrobial resistance. The strains isolated in this study shared a high percentage of core genome with the Swiss strain AE-2096513, also presenting very similar antimicrobial resistance profiles that differed mainly in co-trimoxazole resistance. Interestingly, AE-2096513 was most closely related to HUB11505 and HUB12640, suggesting the spread of this resistant clone in Europe possibly via sexual transmission. Further epidemiological studies are needed to verify the spread of these XDR *H. parainfluenzae* clones.

We identified and characterised a complete capsular operon for the first time in *H. parainfluenzae*. Since the capsule is one of the most important virulence factors in *H. influenzae*, this is a remarkable fact to consider in the surveillance of multidrug-resistant bacterial pathogens. Comparing the predicted genetic structure of the operons, the capsule identified in *H. parainfluenzae* presented the highest identity with that of *H. influenzae* serotype c, which is the least common serotype in this species. The high similarity between their capsules suggests that they might have a common ancestor. AE-2096513 was the only strain out of all those available in the NCBI database that presented a completely identical capsular operon to that of our clinical isolates, which was in accordance with the results of the phylogenetic analysis. Capsule visualisation by TEM indicated that the HUB12445 strain had a similar structure to that of encapsulated *H. influenzae b*, in agreement with previous visualisations of the capsule by TEM^26^ and proving that the capsular operon was expressed in *H. parainfluenzae*. HUB11505, whose *pcsB* gene was interrupted by a transposase, also presented a capsule, but it was morphologically different to the capsule of HUB12445, suggesting that the alternative ORF shown in Fig. 2 encoded a functional protein that modified the polysaccharide composition of the capsule.

In conclusion, this study describes for the first time a capsular operon in *H. parainfluenzae*. The accumulation of resistance mechanisms leaves few therapeutic options in the treatment of these infections. Furthermore, phylogenetic analysis indicates a possible spread of this XDR clone in Europe that could be through sexual transmission.

## Methods

### Characterisation of the bacterial strains

Four XDR *H. parainfluenzae* strains were isolated from the urogenital exudates of three patients between January 2016 and January 2017: two males with urethritis and one female with pelvic inflammatory disease linked to an intrauterine device implantation (Table 1).

One of the male patients presented a positive PCR for *Chlamydia trachomatis*, but was negative for *Neisseria gonorrhoeae*. For the other male, no appropriate samples for PCR detection were collected, with only *H. parainfluenzae* being detected by microbiological culture. The female patient had pelvic inflammatory disease, with *H. parainfluenzae* isolated from both vaginal and endocervical exudates. PCR detection of *C. trachomatis* and *N. gonorrhoeae* gave negative results. *Gardnerella vaginalis* was also isolated from the endocervical exudate.

Clinical isolates were identified as *H. parainfluenzae* by MALDI-TOF mass spectrometry (Bruker Daltonik GmbH, Bremen, Germany). The strains used in this study were routinely grown in chocolate agar plates (bioMérieux, Marcy l’Etoile, France) or *Haemophilus* test medium (HTM) broth (Thermo Fisher Scientific, Massachusetts, USA) and incubated at 37 °C with 5%CO_2_. These strains produced smooth but non-mucoid greyish colonies on chocolate agar plates.

The minimum inhibitory concentration (MIC) was determined by microdilution using commercial panels (STRHAE2; Sensititre, Thermo Fisher Scientific) and interpreted following the guidelines of the European Committee on Antimicrobial Susceptibility Testing (EUCAST)^27^. β-lactamase activity was tested using the chromogenic cephalosporin method (nitrocefin disks, BD, New Jersey, USA).

### Whole-genome sequencing and genome analysis

DNA was extracted using QIAamp DNA Blood Mini Kit (Qiagen, Hilden, Germany) and quantified using the QuantiFluor® dsDNA System (Promega, Wisconsin, USA). Nextera XT was used to prepare the libraries, followed by paired-end sequencing (2 × 150 bp) on an Illumina MiSeq Platform (Illumina Inc., San Diego, CA, USA), following the manufacturer’s instructions. The INNUca v2.6 pipeline (https://github.com/B-UMMI/INNUca) was used for read quality assessment, improvement and genome assembly. Briefly, quality control of the reads was performed using FastQC (http://www.bioinformatics.babraham.ac.uk/projects/fastqc/) and the reads were cleaned and improved with Trimmomatic^28^. The genome was assembled using SPAdes v3.10^29^ and subsequently polished. QA/QC statistics, genome depth of coverage and the number of contigs were monitored and reported throughout the analysis. All generated reads were deposited in the European Nucleotide Archive (accession numbers: HUB11505:ERS2616278, HUB12435:ERS2616279, HUB12445:ERS2616280 and HUB12640:ERS2616281).

An *in silico* screening of mutations targeting genes involved in antibiotic resistance was performed with Geneious R9 (Biomatters, Auckland, New Zealand), using the closed genome of the *H. parainfluenzae* strain T3T1 (NC_015964) as reference. A screening of the acquired resistance genes was conducted using the Comprehensive Antibiotic Resistance Database (CARD)^30^, ARG-ANNOT^31^ and ResFinder 3.0^32^.

Phylogenetic analysis was performed by constructing an assembly-based core-SNV phylogenetic tree including the 35 *H. parainfluenzae* genomes available on the NCBI database and using the T3T1 strain as reference. *Parsnp* from the Harvest suite^33^ was used with default parameters, with the exception of parameter -C, which was adjusted to 5,000 to maximise the reference coverage. Phylogenetic tree visualisation was achieved with the graphic viewer FigTree (http://tree.bio.ed.ac.uk/software/figtree/).

### Capsular operon determination

The capsular operons of *H. influenzae* serotype a (CP017811:1472186-1485236), serotype b (NC_016809:1190909-1206625), serotype c (HQ651151), serotype d (HM770877), serotype e (FM882247) and serotype f (CP005967:675016-687441) were used for a BLASTn search with Geneious R9 against our four assembled genomes and the open reading frames (ORF) were predicted. The percentage of identity between gene/proteins from *H. parainfluenzae* and that of the six capsular operons from *H. influenzae* was calculated as the identical number of bases/residues divided by the length of the longest gene/protein (Supplementary Table 1).

Since the capsular operon of the HUB11505 strain could not be resolved through whole-genome sequencing (WGS) data, we designed the primers 11505_F (5′-GGTTCTACCGACTCGTCTGC-3′) and 11505_R (5′-AGCATCTTCTTGTTTGATATATGCCG-3′) to close the gap by PCR amplification, followed by Sanger sequencing through primer walking, using primers 11505_F and 11505_F2 (5′-GTTCTCGCCTTTGGTTGGCAG-3′). The capsular operon of the *H. parainfluenzae* strain HUB12445 was deposited in the NCBI database with accession number MH644108.

### Transmission electron microscopy for capsule visualisation

Four strains were selected to visualise the presence of the capsule: (a) *H. influenzae* strain HUB13399 serotype b as a positive control; (b) non-encapsulated *H. parainfluenzae* strain HUB13327 as a negative control; (c) *H. parainfluenzae* strains HUB12445 (full *ccsB* gene); and (d) *H. parainfluenzae* strain HUB11505 (*ccsB* gene interrupted by a transposase). Samples were fixed following the lysine-acetate-based formaldehyde-glutaraldehyde ruthenium red-osmium fixation procedure (LRR fixation), as previously described^34^. Strains were grown overnight in HTM broth and centrifuged for 10 min at 1,800 g. Cells were then fixed, dehydrated, impregnated in resin and finally polymerised, as described for *H. influenzae*^26^. Ultra-thin sections were stained with 2% uranyl acetate, counterstained with lead citrate and visualised under JEOL 1010 Transmission Electron Microscope (TEM) operated at 80 kV and equipped with a Orius camera (Gatan, Inc.).

### Ethical statement

This study has been revised and approved for its publication by the Clinical Research Ethics Committee of the Hospital de Bellvitge (PR324/18). Written informed consent was not required as this is a retrospective and observational study with isolates obtained as part of the normal microbiological routine. Patient confidentiality was always protected; all the data were anonymized and protected according to national normative.

## Supplementary information

Supplementary Table 1

## References

[1] Nørskov-Lauritsen N (2014). Classification, identification, and clinical significance of H*aemophilus* and A*ggregatibacter* species with host specificity for humans. Clin. Microbiol. Rev..

[2] Murray, P. R. & Baron, E. J. American Society for Microbiology. *Manual of clinical microbiology*. (ASM Press, 2003).

[3] Kosikowska U, Biernasiuk A, Rybojad P, Łoś R, Malm A (2016). *Haemophilus parainfluenzae* as a marker of the upper respiratory tract microbiota changes under the influence of preoperative prophylaxis with or without postoperative treatment in patients with lung cancer. BMC Microbiol..

[4] Cardines R (2009). *Haemophilus parainfluenzae* meningitis in an adult associated with acute otitis media. New Microbiol..

[5] Latyshev Y, Mathew A, Jacobson JM, Sturm E (2013). Purulent pericarditis caused by *Haemophilus parainfluenzae*. Tex. Heart Inst. J..

[6] O’Neil CR, Wilson E, Missaghi B (2016). Bone and Joint Infections due to *Haemophilus parainfluenzae*: Case Report and Review of the Literature. Can. J. Infect. Dis. Med. Microbiol..

[7] Hong MJ, Kim YD, Ham H (2015). Do. Acute septic arthritis of the acromioclavicular joint caused by *Haemophilus parainfluenzae*: a rare causative origin. Clin. Rheumatol..

[8] Cardines R (2015). Genital carriage of the genus *Haemophilus* in pregnancy: Species distribution and antibiotic susceptibility. J. Med. Microbiol..

[9] Hsu MS, Wu MY, Lin TH, Liao CH (2015). *Haemophilus parainfluenzae* urethritis among homosexual men. J. Microbiol. Immunol. Infect..

[10] Deza G (2016). Isolation of *Haemophilus influenzae* and *Haemophilus parainfluenzae* in urethral exudates from men with acute urethritis: A descriptive study of 52 cases. Sex. Transm. Infect..

[11] Hansson S, Svedhem Å, Wennerström M, Jodal U (2007). Urinary tract infection caused by *Haemophilus influenzae* and *Haemophilus parainfluenzae* in children. Pediatr. Nephrol..

[12] Satola SW, Schirmer PL, Farley MM (2003). Complete sequence of the cap locus of *Haemophilus influenzae* serotype b and nonencapsulated b capsule-negative variants. Infect. Immun..

[13] García-cobos S (2013). Novel mechanisms of resistance to β-lactam antibiotics in *Haemophilus parainfluenzae*: β-lactamase-negative ampicillin resistance and inhibitor-resistant TEM β-lactamases. J. Antimicrob. Chemother..

[14] Abotsi RE, Govinden U, Moodley K, Essack S (2017). Fluoroquinolone, Macrolide, and Ketolide Resistance in *Haemophilus parainfluenzae* from South Africa. Microb. Drug Resist..

[15] Rodríguez-Martínez JM, López-Hernández I, Pascual Á (2011). Molecular characterization of high-level fluoroquinolone resistance in a clinical isolate of *Haemophilus parainfluenzae*. J. Antimicrob. Chemother..

[16] Tinguely R (2013). Emergence of extensively drug-resistant *Haemophilus parainfluenzae* in Switzerland. Antimicrob. Agents Chemother..

[17] Magiorakos AP (2012). Multidrug-resistant, extensively drug-resistant and pandrug-resistant bacteria: An international expert proposal for interim standard definitions for acquired resistance. Clin. Microbiol. Infect..

[18] Endimiani A, Allemann A, Wüthrich D, Lupo A, Hilty M (2017). First report of the macrolide efflux genetic assembly (MEGA) element in *Haemophilus parainfluenzae*. Int. J. Antimicrob. Agents.

[19] Faccone D (2016). Molecular characterization of a clinical *Haemophilus parainfluenzae* isolate with cefotaxime resistance and decreased susceptibility to fluoroquinolones. Infect. Genet. Evol..

[20] Tristram SG (2008). Characterization of extended-spectrum β-lactamase-producing isolates of *Haemophilus parainfluenzae*. J. Antimicrob. Chemother..

[21] Law DKS, Shuel M, Bekal S, Bryce E, Tsang RSW (2010). Genetic detection of quinolone resistance in *Haemophilus parainfluenzae*: Mutations in the quinolone resistance-determining regions of *gyrA* and *parC*. Can. J. Infect. Dis. Med. Microbiol..

[22] Georgiou M (1996). Ciprofloxacin-resistant *Haemophilus influenzae* strains possess mutations in analogous positions of GyrA and ParC. Antimicrob. Agents Chemother..

[23] Peric M, Bozdogan B, Jacobs MR, Appelbaum PC (2003). Effects of an efflux mechanism and ribosomal mutations on macrolide susceptibility of *Haemophilus influenzae* clinical isolates. Antimicrob. Agents Chemother..

[24] de Groot R (1996). Genetic characterization of trimethoprim resistance in *Haemophilus influenzae*. Antimicrob. Agents Chemother..

[25] Varaldo PE, Montanari MP, Giovanetti E (2009). Genetic elements responsible for erythromycin resistance in streptococci. Antimicrob. Agents Chemother..

[26] Schouls L (2008). Two variants among *Haemophilus influenzae* serotype b strains with distinctb*cs4*, h*csA a*nd h*csB g*enes display differences in expression of the polysaccharide capsule. BMC Microbiol..

[27] The European Committee on Antimicrobial Susceptibility Testing. Clinical breakpoints. European Society of Clinical Microbiology and Infectious Diseases (EUCAST) Basel, Switzerland. Available at, http://www.eucast.org/clinical_breakpoints/ (2018).

[28] Bolger AM, Lohse M, Usadel B (2014). Trimmomatic: A flexible trimmer for Illumina sequence data. Bioinformatics.

[29] Bankevich A (2012). SPAdes: A New Genome Assembly Algorithm and Its Applications to Single-Cell Sequencing. J. Comput. Biol..

[30] Jia B (2017). CARD 2017: Expansion and model-centric curation of the comprehensive antibiotic resistance database. Nucleic Acids Res..

[31] Gupta SK (2014). ARG-annot, a new bioinformatic tool to discover antibiotic resistance genes in bacterial genomes. Antimicrob. Agents Chemother..

[32] Zankari E (2012). Identification of acquired antimicrobial resistance genes. J. Antimicrob. Chemother..

[33] Treangen TJ, Ondov BD, Koren S, Phillippy AM (2014). The harvest suite for rapid core-genome alignment and visualization of thousands of intraspecific microbial genomes. Genome Biol..

[34] Hammerschmidt S (2005). Illustration of Pneumococcal Polysaccharide Capsule during Adherence and Invasion of Epithelial Cells. Infect. Immun..

