# Supplementary material for: Identification of polysaccharide capsules among extensively drug-resistant genitourinary Haemophilus parainfluenzae isolates
Source: Sci Rep. 2019 Mar 14;9:4481. doi: 10.1038/s41598-019-40812-2 (PMC6418240; doi:10.1038/s41598-019-40812-2)
Supplement: Supplementary file 1 — Supplementary Table 1 [file 41598_2019_40812_MOESM1_ESM.pdf]

Identification of polysaccharide capsules among extensively drug-resistant genitourinary *Haemophilus parainfluenzae* isolates

Aida González-Díaz[1,2], Fe Tubau[1,2], Miguel Pinto[3], Yanik Sierra[1], Meritxell Cubero[1,2], Jordi Càmara[1,2], Josefina Ayats[1,2], Paula Bajanca-Lavado[4], Carmen Ardanuy[1,2]*, Sara Marti[1,2]*.

(A) Gene

	bexA		*bexB*		*bexC*		*bexD*		*xcsA*		*xcsB*		*xcsC*		*xcsD*		*hcsA*		*hcsB*	
	Identity (%)	Length (bp)	Identity (%)	Length (bp)	Identity (%)	Length (bp)	Identity (%)	Length (bp)	Identity (%)	Length (bp)	Identity (%)	Length (bp)	Identity (%)	Length (bp)	Identity (%)	Length (bp)	Identity (%)	Length (bp)	Identity (%)	Length (bp)
**HPAR[a]**		**654**		**798**		**1134**		**1185**		**939**		**3717**		**1230**		**1122**		**2043**		**1221**
**Hia**	86.5	654	85.2	798	85.1	1134	91.2	1185	21.6	1425	9.9	1116	35.6	2340	12.8	381	94.2	2043	94.2	1227
**Hib**	53.5	654	85.5	798	91.1	1134	91.1	1185	21.7	1425	10.3	1116	35.1	3648	21.1	1848	94.4	2043	94.4	1227
**Hic**	NA	NA	NA	NA	NA	NA	NA	NA	91.0	939	58.6	3672	95.6	1230	96.7	1118	NA	NA	NA	NA
**Hid**	NA	NA	NA	NA	NA	NA	NA	NA	27.7	1125	11.4	1263	35.9	2943	20.0	1968	NA	NA	NA	NA
**Hie**	91.2	654	95.2	798	97.0	1134	87.8	1287	30.8	1125	11.8	1266	33.5	2976	27.1	1461	82.7	1788	91.2	1263
**Hif**	89.7	654	95.1	798	97.1	1134	95.4	1185	78.7	1095	25.5	2664	32.2	1140	-	-	94.9	2043	89.9	1221

(B) Protein

	BexA		BexB		BexC		BexD		XcsA		XcsB		XcsC		XcsD		HcsA		HcsB	
	Identity (%)	Length (AA)	Identity (%)	Length (AA)	Identity (%)	Length (AA)	Identity (%)	Length (AA)	Identity (%)	Length (AA)	Identity (%)	Length (AA)	Identity (%)	Length (AA)	Identity (%)	Length (AA)	Identity (%)	Length (AA)	Identity (%)	Length (AA)
**HPAR[a]**		217		265		377		395		313		1239		410		374		681		407
**Hia**	97.2	217	93.5	265	90.9	377	93.4	395	<1	313	<1	372	<1	780	<1	127	95.7	681	87.6	409
**Hib**	97.2	217	94.7	265	94.1	377	93.0	395	<1	475	<1	372	<1	1216	<1	616	96.1	681	88.7	409
**Hic**	NA	NA	NA	NA	NA	NA	NA	NA	91.3	475	48.5	1224	95.8	410	TRUNCATED		NA	NA	NA	NA
**Hid**	NA	NA	NA	NA	NA	NA	NA	NA	<1	313	<1	421	<1	981	<1	656	NA	NA	NA	NA
**Hie**	96.3	217	97.7	265	97.1	377	96.7	429	<1	375	<1	422	<1	992	<1	487	83.7	596	90.9	421
**Hif**	95.9	217	97.3	265	98.4	377	97.9	395	76.5	375	20.0	888	<1	380	-	-	96.3	681	90.1	407

Supplementary Table 1. Capsular operon identity. The number of identical bases/amino acids divided by the length of the longest gene (A) and protein (B) between genes/proteins from *H. parainfluenzae* and those from the six *H. influenzae* serotypes are expressed in percentages. Gene length is expressed in base pairs (bp) and protein length in the number of amino acids (AA).

[a] Capsular operons from *H. influenzae* serotypes a-f are abbreviated as Hia-Hif, while that from *H. parainfluenzae* is abbreviated as HPAR.

NA, no available sequence in NCBI. -, gene/protein does not exist in the operon.
